# Congenital Complete Heart Block—To Stimulate (When?) or Not to Stimulate?

**DOI:** 10.3390/healthcare12121158

**Published:** 2024-06-07

**Authors:** Piotr Kukla, Beata Podlejska, Jerzy Wiliński

**Affiliations:** 1 Department of Internal Medicine and Cardiology, Specialist Hospital H. Klimontowicza, 38-300 Gorlice, Poland; podlejska.b@gmail.com; 2 Center for Invasive Cardiology, Electrotherapy and Angiology, 33-300 Nowy Sącz, Poland; putamen@interia.pl; 3 Department of Internal Medicine with Cardiology Subdivision, Blessed Marta Wiecka District Hospital, 32-700 Bochnia, Poland

**Keywords:** congenital heart block, cardiac pacemaker, atropine, salbutamol

## Abstract

This article presents the case of a 27-year-old female patient with idiopathic congenital complete heart block who does not consent to the implantation of a cardiac pacemaker but was referred by her primary care physician for cardiological evaluation. The conduction disturbance was recognized at the age of 6 and was asymptomatic. The professional disqualification from pacemaker implantation included a detailed history of a patient’s symptoms, an echocardiographic assessment of the heart, exercise testing and ECG Holter monitoring. The aid of salbutamol administered orally was also useful.

## 1. Introduction

Complete heart block (CHB), also known as third-degree atrioventricular block (AV III block), is a disturbance with abnormal propagation of the electrical impulses due to structural or functional abnormalities of the cardiac conduction system at the level of the AV junction or below it. CHB might occur in a structurally normal heart as an isolated disorder or might accompany various congenital heart defects [1]. CHB is classified as congenital AV block if it is diagnosed in utero, at birth or within the first month of life. Childhood AV block diagnosis is made when CHB is identified between the first month and eighteenth year of life [2]. In general, the incidence of third-degree congenital AV heart block is 1 in every 15,000–22,000 live births [1,3,4]. CHB, as a complication of an autoimmune process, is characterized by high neonatal mortality rates, whereas the overall mortality rate without cardiac pacing is 8–16% in infants and is half this rate in children and adults [5,6].

Apart from a diversified etiology, CHB is associated with different clinical presentations. Thus, patients may be asymptomatic or present with reduced exercise capacity, syncopal attacks and symptoms of heart failure related to bradycardia [2]. Nevertheless, imaging studies can reveal the alterations of atrial and ventricular myocardium associated with proinflammatory states with the signs of myocarditis and endocardial fibroelastosis. This would lead to atrial and ventricular dilatation; hyperechogenicity, especially of the atrial walls, due to fibrosis and reduced ventricular contractility with clinical complications of chronic heart failure with pericardial effusion; ascites or fetal hydrops [7,8]. Moreover, all these pathological phenomena can promote diverse heart arrhythmias. Although sudden cardiac death is rare, bradycardia itself may predispose one to dangerous ventricular arrhythmias such as the polymorphic ventricular tachycardia called torasade-de-pointes [9].

If the heart block is transient, usually no further therapy is required. Whenever congenital CHB is permanent, cardiac pacing should be considered even in children, if there are no contraindications [10]. On the other hand, as in many centers, all patients with CHB are qualified for a pacemaker implantation; the question thus arises, does literally everyone with congenital CHB require permanent cardiac stimulation?

A doctor consulting or caring for a patient with congenital AV block always has concerns about whether or not to refer the patient for pacemaker implantation (“pace or not-to-pace”). Thus, which essential tools, apart from a resting electrocardiogram (ECG), need to be used to assess a patient for indications of pacemaker implantation?

1. A detailed medical history needs to be collected in order to answer the question of whether there are any symptoms (syncope, exercise intolerance, palpitations and symptoms of heart failure).
2. A physical examination ought to check the signs of bradycardia and heart failure.
3. Echocardiography should be performed to assess the presence of valvular heart disease and to assess the size and function of the heart. In some patients, long-term AV block may cause left ventricular dyssynchrony.
4. An exercise ECG should be performed and chronotropic capacity and exercise tolerance should be assessed.
5. ECG monitoring should use the Holter method to assess the average, minimum and maximum rhythm rates and to assess whether there is ventricular arrhythmia, inhibitions and interruptions in the leading rhythm and QT interval duration [11].

## 2. Case Presentation

### 2.1. Medical History

We present the case of a 27-year-old female patient with congenital CHB who was referred by her primary care physician for cardiological evaluation. The complete AV block was recognized when she was 6 years old. Neither structural abnormalities of the heart were found nor could autoimmune diseases be confirmed in the diagnostics performed at the time of diagnosis and in the subsequent follow-up. The idiopathic CHB was recognized. Ever since, the patient has been asymptomatic and denied all the above-mentioned symptoms, which were the main reasons why her parents did not consent to the girl having a pacemaker implanted; neither did the patient herself. Additionally, there was no history of other chronic conditions requiring pharmacological treatment and no family history of heart diseases and conduction problems.

### 2.2. Diagnostics

The physical examination showed no signs of heart failure and only a slow pulse of 50 bpm. The blood pressure was 115/70 mm Hg.

**Figure 1:**
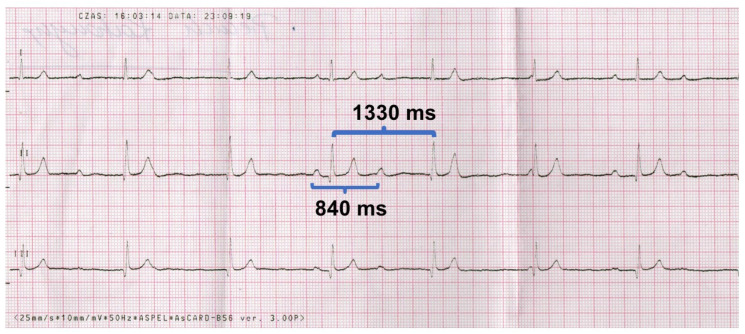
Resting electrocardiograms at standard paper speed of 25 mm/s and normal calibration of 1 mV = 10 mm. Limb leads I–III, sinus rhythm cycle 840 ms (71 bpm), junctional rhythm cycle 1330 ms (45 bpm).

**Figure 2:**
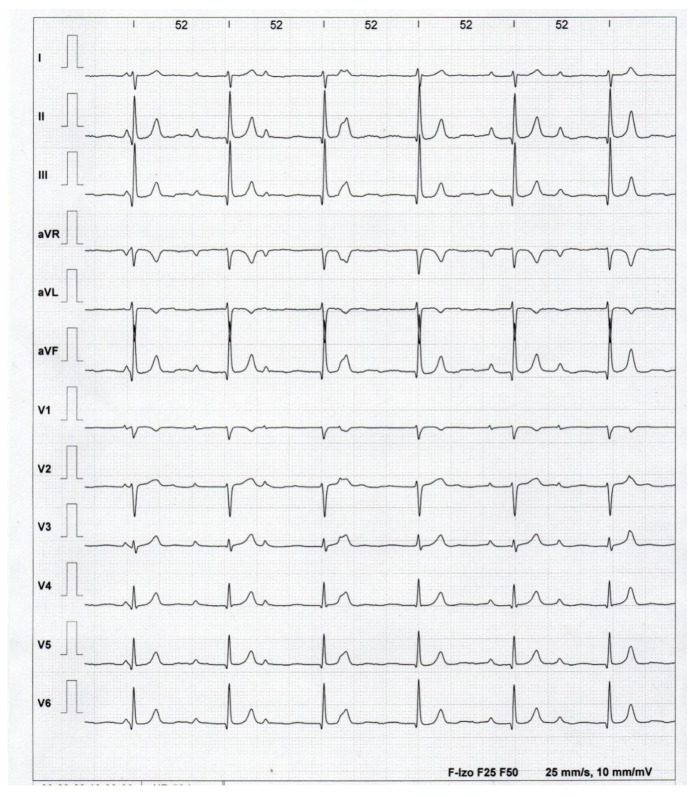
ECG before exercise test. Sinus rhythm 68/min and AV link rhythm 52/min.

**Figure 3:**
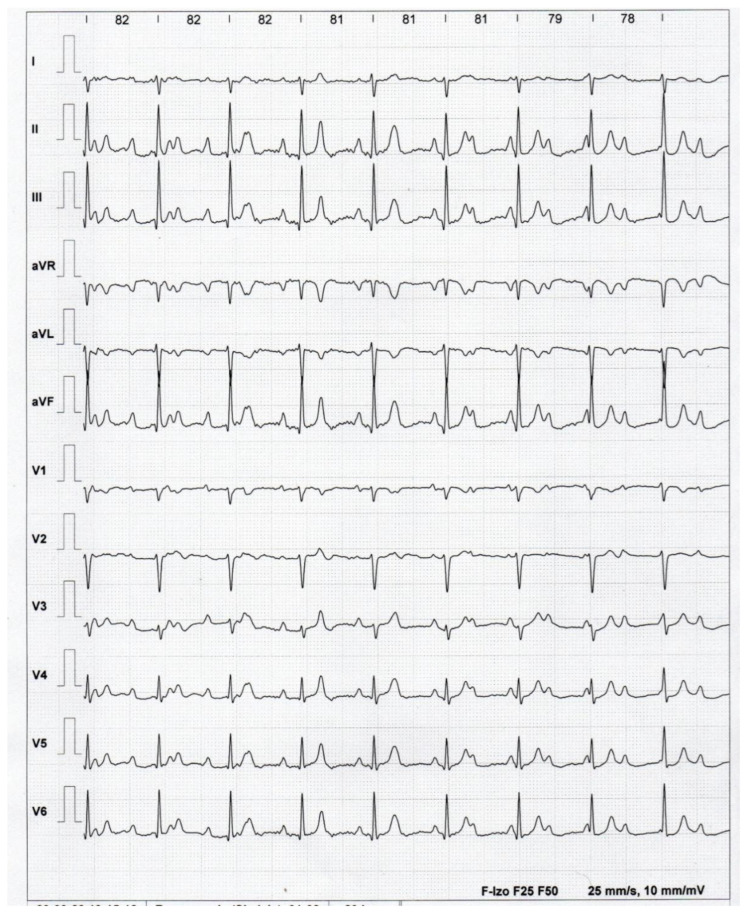
ECG at the top of the exercise test. The sinus rhythm accelerated to 136/min (cycle 440 ms), and the junctional rhythm accelerated to 82 bpm.

The ECG recording is shown in Figure 1, Figure 2 and Figure 3. We have two separate rhythms: the sinus rhythm of the atria (sinus P waves visible) at approximately 70 beats per minute (bpm), and the AV link rhythm at 45 bpm (cycle’s length of 1330 ms). The QT interval is 440 ms; the corrected QT interval-QTc is 380 ms.

Transthoracic echocardiography (Vivid S60N, General Electric Company, Boston, MA, USA) showed normal function (left-ventricular ejection fraction-EF of 65%), normal sizes of the heart chambers and the atrioventricular valves’ function. The treadmill exercise test performed according to Bruce’s protocol (TRM-612 Cardiotest, v.702ALF, Aspel SA, Zabierzów, Poland), without medical treatment in the 48 h preceding the examination, showed good chronotropic efficiency with an acceleration of the heart rate from 45 bpm to approximately 80 bpm. The patient had a very good exercise tolerance of 12.4 metabolic equivalents (METs), without any disturbing clinical symptoms. Holter ECG monitoring (BTL-08 Holter, H300, BTL Corporate, Ashford, UK) showed a mean heart rate with a junctional rhythm of 54 bpm (minimal heart rate 41 bpm, maximal heart rate 78 bpm; the patient was using atropine sulfate 0.25 mg once daily and salbutamol 4 mg once daily orally). The mean atrial sinus rhythm was 74 bpm. No supraventricular or ventricular arrhythmias were recorded. There were no pauses above 3 s.

### 2.3. Management

Therefore, after the initial diagnosis, there were no reasons to recommend the patient for pacemaker implantation. So far, the patient has been using the beta-2 and beta-1 adrenergic receptor-agonist salbutamol 4 mg once daily and tropane alkaloids (atropine sulfate 0.25 mg) once daily orally. We have modified the treatment. Only 4 mg of salbutamol was taken once daily orally.

### 2.4. Follow-Up

During the 18 months of observation, the patient remained asymptomatic and required no medical assistance.

## 3. Discussion

In children and adolescents, CHB associated with an autoimmune disease, predominantly systemic lupus erythematosus or Sjoegren’s syndrome, is the most common form. The congenital CHB associated with neonatal lupus is considered a form of passively acquired autoimmune disease with maternal autoantibodies to the intracellular ribonucleoproteins Ro (SS-A) and La (SS-B). They cross the placenta and through fetal circulation bind to L-type calcium channels of cardiomyocytes which induces the perturbation of calcium metabolism, leading to the apoptosis of cells, local inflammation and fibrosis of the cardiac conduction system [12]. Women with serum titers of anti-Ro antibodies carry a 3% risk of having a child with neonatal lupus syndrome. Recurrence rates are about 18% [13]. Interestingly, only 2–5% of pregnancies with elevated circulating maternal anti-Ro/SSA or anti-La/SSB antibody levels result in fetuses with CHB [3].

Secondary CHBs are one of the main complications of congenital heart disease surgery [14]. The most common structural abnormalities involve the transposition of the great vessels and the defects of the AV septum [15]. It is noteworthy that in many patients, the cause of third-degree congenital AV block is unknown. These idiopathic forms of CHB might be associated with genetic abnormalities without apparent associations with autoimmune diseases and observed in a structurally normal heart [2]. CHB might have the background of complex pathophysiological processes related to certain genetic variants of genes and gene network coding ion channels, including SCN5A, SCN1B, SCN10A, TRPM4, KCINK17, CAVB, KCNJ2, HCN4, LMNA, ANKB, NKX2-5, TBX5, and cardiac connexin proteins, leading to isolated AV block or to progressive cardiac conduction disease with various intraventricular conduction disturbances [1,16,17]. Moreover, inherited progressive cardiac conduction disease (PCCD) with interventricular and AV conduction disturbances and subsequent CHB has been identified as associated with mutations of genes encoding for the proteins crucial for cardiac chamber formation, endocardial cushion remodeling and conduction system development [18].

CHB in children and young adults can occur as a consequence of a broad array of causes, such as infectious diseases with myocarditis, infiltrative processes, metabolic abnormalities, hypothyroidism, pathological neurocardiogenic mechanisms or even coronary artery disease. It can be also seen in Lyme carditis, Chagas disease, the inflammatory conditions of Kawasaki disease and acute rheumatic carditis [19,20]. CHB is among the rare but typical complications of surgical and catheterization-induced trauma, interventional procedures of ventricular septum defects’ corrections and catheter ablations of AV nodal reentrant tachycardia and septal accessory pathways, especially of parahisian localization [2,21].

Throughout recent decades, there has been a common belief that all patients with congenital CHB, even the asymptomatic ones, should have a pacemaker implanted when first diagnosed [6,22]. This stemmed from the results of studies on children born from the 1970s to 1990s [23]. Later analyses revealed that not every patient with congenital CHB needs a permanent cardiac pacing system [11,24].

Thus, what are the indications for permanent pacing in adult patients with congenital AV block? According to the current European Society of Cardiology’s 2021 guidelines on cardiac pacing, the indication for permanent cardiac pacing in class I recommendations is the presence of one of the following risk factors of the aforementioned CHB complications:

1. The occurrence of the symptoms mentioned earlier;
2. Left ventricular systolic dysfunction;
3. A number of pauses > 3 times the basic rhythm (e.g., in our case the average cycle is 1330 ms, so the pause > 3.990 ms (>~4 s);
4. An escape rhythm with wide QRS complexes;
5. A prolonged QTc interval; it should be remembered that Bazett’s correction rule overestimates QTc for rhythms above 100 bpm and underestimates QTc for rhythms below 50 bpm;
6. The occurrence of complex ventricular arrhythmias;
7. An average daily heart rate < 50 bpm [11,25,26].

Thus, after determining the cause of the AV block, every symptomatic and non-reversible CHB and asymptomatic CHB with the aforementioned risk factors requires permanent pacemaker implantation [10,11]. This is certainly the case in the absence of contraindications to the procedure, including severe coagulation abnormalities, excessive risk of bleeding due to vascular access, local infection at the implantation site or ongoing bacteremia. There is also an issue of the patient’s consent or the consent of parents of an underage patient. At times, aesthetic reasons are responsible for the lack of consent for implantation, especially in women [27]. Additionally, another reason for refusal may be the fear of complications. The most common complications of pacemaker implantation procedure involve lead-related reinterventions (including dislodgement, malposition, subclavian crush syndrome), implantable electronic devices, (CIED)-related infections (superficial infection, pocket infections and systemic infections), pneumothorax, hemothorax, brachial plexus injury, cardiac perforation, coronary sinus dissection/perforation, the need for revision due to pain/discomfort, diaphragmatic stimulation requiring reintervention, hematoma, tricuspid regurgitation, pacemaker syndrome, a generator/lead problem, deep venous thrombosis (acute or chronic) and death [11]. In a real-life study on >81,000 adult recipients of cardiac-implantable electronic devices, major complications occurred in 8.2% of patients within 90 days of hospital discharge [28]. The scale of the problem in the pediatric population is significantly greater [29].

Classic right-ventricular stimulation with a right-ventricular apex position of the lead tip is easily accessible and provides a stable position. Unfortunately, it has been shown to be complicated by ventricular desynchronization, myocardial dystrophic changes and cardiac dilatation, and is recognized as an important cause of dilated cardiomyopathy (pacing-induced cardiomyopathy) [8,30]. Techniques of cardiac pacing have been evolving throughout the years to allow a safer and more efficient performance of the heart, even in patients with heart defects and after corrective surgeries. Some of these complications could be significantly reduced by the implementation of cardiac resynchronization therapy (CRT), with an extra lead placed in the coronary sinus tributary responsible for the stimulation of the lateral and posterior walls of the left ventricle, at the same time as the interventricular septum activation is evoked by the right ventricular lead [31,32]. In very recent years, we have also witnessed the flourishing of methods based on conduction system pacing (CSP), directly encompassing the His bundle and its branches’ stimulation. CRT and CSP, together referred to as cardiac physiologic pacing (CPP), are widely propagated in order to avoid or mitigate heart failure development related to permanent pacing [33].

Cardiac pacing in pediatric patients encounters its distinctive problems in relation to anatomy and somatic growth. Although the smallest infants, weighing below 15–20 kg, are served best with epicardial systems, epicardial leads are susceptible to fracture and exit-block development and require a major operation. On the contrary, endocardial systems are associated with increased venous thrombosis in infants, which may result in the loss of venous access. Moreover, the extraction of transvenous leads in children is more difficult and bears a higher risk of periprocedural complications. Nevertheless, the longevity of endocardial systems, especially with spare lead loops, exceeds the endurance of epicardial ones [34,35]. As the transvenously implanted leads are considered the Achilles’ heel of pacemakers, and after years they need to be replaced with new ones, great hopes are associated with leadless systems. Advances in battery technology and the miniaturization of electronics now offer the opportunity to implant the whole pacemaker system into the right ventricle in selected groups of patients, e.g., without venous access. The latest models are even equipped with an AV synchronization function based on a sensor detecting the mechanical movements of atria [36]. It also seems that new methods introduced in the treatment of bradycardia such as cardioneuroablation may find application in some patients with AV blocks [37].

The female patient who presented with applied pharmacology had none of the risk factors; thus, pacemaker implantation could be avoided. Nevertheless, constant observation and repeated evaluations of these risk factors on a regular basis is necessary.

The role of the pharmacological treatment of CHB is limited. Tracing the natural history of CHB development in collagen tissue diseases, its onset commonly occurs between 18 and 24 weeks of gestational age [38,39]. It is frequently preceded by ventricular ectopy, junctional and ventricular tachycardia, changes of ST-T and QT interval prolongation. The fetus then adopts bradycardia, usually of 50–70 bpm. Nevertheless, on some occasions, the heart rate drops below 50 bpm and displays low variability. In these cases, a beta-adrenergic agonist terbutaline might be used to increase heart rate. Additionally, the use of dexamethasone for CHB in some centers was linked to reduced long-term cardiomyopathy. Intravenous immunoglobulin has been shown to prevent mortality in fetuses with hydrops. It also reduced endocardial fibroelastosis and subsequent ventricular dysfunction. Moreover, dexamethasone or betamethasone and hydroxychloroquine hampered progression in partial AV block to CHB in systemic lupus erythematosus [39,40]. In some centers, prenatal treatment, apart from intravenous immunoglobulins, also includes plasmapheresis and their combination with steroids [41,42]. In these regimens, the side effects of the drugs need to be considered. The significant side effects of fluorinated steroids are miscarriage, oligohydramnios, delayed development and growth retardation and adrenal insufficiency. The therapeutic complications in mothers involving diabetes, hypertension and weight gain might be significant [1].

After birth, bradycardia (usually of <70 bpm) can initially be controlled by drugs such as isoprenaline, atropine, epinephrine and dopamine, alone or in combination with temporary cardiac pacing to prevent sudden death and before permanent pacing introduction [2,43,44]. It is noteworthy that, even in asymptomatic neonates or infants, permanent pacemaker implantation is indicated when the mean ventricular rate is ≤50 bpm. Nevertheless, ventricular rate alone in children should not be used as implant criteria. The symptoms of bradycardia are a crucial aspect of qualification for permanent pacing. Symptoms related to low cardiac output may occur even at faster heart rates than 50 bpm [10].

As for the pharmacological treatment of adult patients with CHB, which is rather an alternative approach, data are scarce and come on a case-by-case basis. The drugs used there decrease vagal nerve activity, like atropine and its derivative, or stimulate the sympathetic nervous system like salbutamol [45]. There are also reports of methylxanthines-theophylline and aminophylline (a theophylline derivative) having positive effects on CHB [46]. The oral preparation of atropine sulfate is registered in Poland to treat spasms in the gastrointestinal tract (gastric colic, intestinal colic), urinary tract and biliary tract conditions in which it is necessary to reduce the secretion of saliva and bronchial secretions. For these indications, one to two tablets are used two to three times a day. When they are used as chronotropic-positive agents, lower doses are usually utilized. The oral agent of salbutamol is used to treat asthma, bronchospasms and/or reversible airway spasms. Its dosage covers 2–4 mg taken three to four times a day. Again, reduced doses are used in the treatment of bradycardia. Slow-release preparations of Theophylline are available on the market to prevent bronchial spasms in bronchial asthma and chronic obstructive pulmonary disease with dosages of 150–900 mg. In all these drugs, the minimum effective dose is applied to treat bradycardia in order to avoid the side effects of active substances [45].

## 4. Conclusions

Congenital complete heart block is a condition that potentially requires pacemaker implantation. Repeated careful risk stratification with the exclusion of the risk factors of health- and life-threatening complications of complete heart block might help to avoid permanent heart pacing introductions. The aid of chronotropic-positive drugs might be helpful in this scenario.

## Data Availability

Data are contained within the article.
